# Reasons for Polytobacco Use among Young Adults: Scale Development and Validation

**DOI:** 10.18332/tpc/64238

**Published:** 2016-07-20

**Authors:** Carla J. Berg, Regine Haardörfer, Gillian Schauer, Getachew Betelihem, Matthew Masters, Bennett McDonald, Jill Shah, Jackelyn Payne, Raina Sarmah, Matthew Thomas, Michael Windle

**Affiliations:** a Department of Behavioral Sciences and Health Education, Rollins School of Public Health, Emory University, 1518 Clifton Rd NE, Atlanta, GA 30322; b Battelle Public Health Center for Tobacco Research, 1100 Dexter Avenue North, Suite 400 Seattle, WA 98109-3598; c Department of Epidemiology, Rollins School of Public Health, Emory University, 1518 Clifton Rd NE, Atlanta, GA 30322; d Department of Health Policy and Management, Rollins School of Public Health, Emory University, 1518 Clifton Rd NE, Atlanta, GA 30322; e ICF Macro, 1725 I (Eye) Street NW, 10th Floor, International, Washington, DC 20006

**Keywords:** Substance use, Young adults, Risk factors, Tobacco use

## Abstract

**INTRODUCTION:**

Limited research has examined reasons for polytobacco use, an increasing public health problem, particularly among young adults. We examined reasons for polytobacco use among users of more than one tobacco product in the past 4 months enrolled in an ongoing six-wave longitudinal study of 3,418 students aged 18-25 from seven US colleges and universities.

**METHODS:**

An expert panel generated items related to reasons for polytobacco use, included in Wave 3 (administered in Summer 2015). Participants reporting use of more than one tobacco product in the past four months (n=540) were asked to complete the Reasons for Polytobacco Use scale and measures related to tobacco/nicotine use/dependence, use motives, perceptions of tobacco, parental/friend use, other substance use, and mental health. We conducted a factor analysis and then examined convergent and discriminant validity for the derived factors.

**RESULTS:**

Our sample was an average age of 20.40 (SD=1.84), 48.0% male, and 21.9% Black. Four factors were identified: Instrumentality, Social Context, Displacement, and Experimentation. Instrumentality was the only factor associated with little cigar/cigarillo and marijuana use. Displacement and Social Context showed similar associations; however, Social Context was associated with having friends who used tobacco while Displacement was not. Experimentation was associated with greater perceived addictiveness and harm of using tobacco products as well as greater perceived social acceptability of tobacco use.

**CONCLUSIONS:**

Each of the four factors identified demonstrated unique convergent and discriminant validity. The use of this scale to characterize polytobacco using young adults may help inform and target cessation or prevention interventions.

## INTRODUCTION

While traditional cigarettes continue to be the main source of tobacco use in the US,^1, 2^ various alternative tobacco products (ATPs), including little cigars and cigarillos (LCCs), smokeless tobacco (SLT), electronic cigarettes (e-cigarettes), and hookah, have recently been introduced to the US market, with use and awareness of these products dramatically increasing, particularly among young adults^3-5^. ATP use represents health risks. For example, LCCs can deliver sufficient amounts of nicotine to maintain dependence and can cause several chronic diseases (e.g., coronary heart disease, lung diseases, cancer)^6^. Additionally, although e-cigarettes represent promise for harm reduction in smokers,^7-11^ research has documented that e-liquids contain detectable levels of carcinogens (formaldehyde, certain tobacco-specific nitrosamines) and toxins (diethylene glycol),^12, 13^ and e-cigarette use has adverse pulmonary effects^14^. Furthermore, hookah use produces carbon monoxide, nicotine, tar, and heavy metals at levels similar to or higher than cigarettes^3^.

ATPs have significantly altered the terrain of tobacco use, particularly among young adults (i.e., those aged 18-24 years). Per the 2012-2013 National Adult Tobacco Survey, current use prevalence in this population was: 18.5% cigarettes, 3.4% LCCs, 4.4% SLT, 2.4% e-cigarettes, and 2.5% hookah^15^. Of particular relevance to the current study, recent research has documented high rates of polytobacco use, particularly in this population^16-18^. Roughly 15-30% of young adult smokers currently use more than one tobacco product;^19, 20^ among ATP users, polytobacco use has increased to 40-50%^21, 22^. Beyond the risks of using a single ATP, polytobacco use may increase risk of nicotine dependence^23-25^.

The reasons for polytobacco use are not well known. One major reason for using various substances concurrently may be to achieve the synergistic effects of substances used simultaneously^26, 27^. This may hold true both within the tobacco product category and outside the range of tobacco products (e.g., marijuana)^28^. Another possible reason for polytobacco use may be that some are more socially appealing than others. For example, hookah use is seen as particularly socially acceptable,^29-31^ whereas other products such as cigarettes are not^32^. Thus, people may prefer the use of one tobacco product over another in certain social settings. Additionally, some tobacco products might be used to reduce or quit the use of another or to circumvent smoke-free policies^33^. Finally, experimentation is another possible reason for polytobacco use, particularly among young adults^34^. Notably, many ATPs, particularly e-cigarettes and hookah, are often perceived as less harmful and addictive among young adults, thus increasing the likelihood of experimentation and uptake^32^.

Drawing from the Theory of Planned Behavior^35, 36^ and Social Cognitive Theory^37^, several individual and sociocontextual characteristics may be distinctly associated with different reasons for polytobacco use. For example, more favorable attitudes toward use or lower perceived risk of tobacco use may be related to polytobacco use for purposes of achieving synergistic effects, displacement, or experimentation. Moreover, social environment and subjective norms may play a role in polytobacco use. Those with parents, friends, and other social influences who use tobacco may perceive social norms that are more conducive to tobacco use and may be more sensitive to social context^32, 38^. New tobacco products may also be more available to them for experimentation^32, 38^. Additionally, polytobacco use might be associated with polysubstance use and a genetic propensity for addiction in general, particularly if a main reason for polytobacco use is to achieve the synergistic effects of substances^32, 38^. Furthermore, individual mental health may differentially impact reasons for polytobacco use. For example, depressive symptoms may be associated with polytobacco use among heavier users who may be self-medicating. Depressive symptoms, however, may not be associated with experimentation^38-41^. Finally, certain reasons for polytobacco use might be associated with use of specific tobacco or nicotine products or with higher levels of addiction.

In summary, there has been an increase in polytobacco use, but limited research, particularly quantitative research, has been conducted to assess the context and reasons for polytobacco use. Assessing the reasons for polytobacco use and examining correlates of these reasons may inform intervention efforts. Thus, the current study aimed to develop and test the reliability and validity of a scale to assess reasons for polytobacco use among young adults reporting the use of more than one tobacco or nicotine product in the past four months.

## METHODS

### Participants and Procedures

The parent study, entitled Project DECOY (Documenting Experiences with Cigarettes and Other Tobacco in Young Adults), was approved by the Emory University and ICF International Institutional Review Boards as well as those of the participating colleges. Project DECOY is a sequential mixed-methods^42^ longitudinal panel study of 3,418 college students from seven colleges in Georgia. Note that, while recent estimates of use prevalence of LCCs, e-cigarettes, and hookah among young adults in Georgia are not available, 2014 CDC Behavioral Risk Factor Surveillance data^43^ indicated a prevalence of current cigarette and SLT use of 16.4% and 5.4%, respectively. The colleges in this study included two public universities/colleges, two private universities, two community/technical colleges, and a historically black university located in rural and urban settings. Detailed information on this study is provided elsewhere^44^ but briefly summarized here.

Contact information (e.g., email addresses) was obtained from the registrar's office from each college/university for students meeting eligibility criteria (i.e., ages 18-25; able to speak English). Three thousand randomly selected 18-25 year olds were selected from one private and two public universities. The remainder of the schools had 18-25 year old student populations of less than 3,000; thus, the entire student population of that age range at those schools was included in recruitment. Response rates ranged from 15.4% to 27.6% at the technical colleges; 12.0% and 19.2% at the public colleges/universities; 18.8% and 59.4% at the private universities; and 23.1% at the historically black university. Our overall response rate of 22.9% (N=3,574/15,607), albeit low, was over a very short time frame (24 hours at the private schools to seven days at the technical colleges) and met our sampling quota targets^44^. Our intent was to enroll participants who were engaged in email and were potentially more likely to be retained in the subsequent waves of the larger, multi-wave longitudinal project. The sociodemographics characteristics of the baseline sample was largely reflective of the student bodies of the college campuses included in this study; however, our sample was disproportionately female^44^.

Data collection began in Fall 2014 and consists of self-report assessments via an online survey every four months for two years (during Fall, Spring, and Summer). Current analyses draw from the baseline assessment of sociodemographic information and Wave 3 data collected in Summer 2015. Retention at Wave 3 was 83.9% (n=2869/3418). In order to ensure that we had sufficient sample size, we directed all participants who used more than one tobacco product in the past 4 months (a time frame selected to cover the period of time between assessments) to the Reasons for Polytobacco Use section. At Wave 3, 1,087 (37.9%) of participants used any tobacco in the past 4 months (786 [27.4%] of all participants used any tobacco in the past 30 days). For the current analysis, we included the 540 participants who used more than one tobacco product in the past 4 months (18.8% of the total; 49.7% of tobacco users). Notably, 221 (40.9% of polytobacco users) used two tobacco products, 189 (35.0%) used three, and 130 (24.1%) used four or more.

### Measures

Data collected at Wave 3 included a range of psychosocial and substance use variables. Below we outline our primary measure of focus – the newly developed Reasons for Polytobacco Use Scale – and the correlates of interest.

#### Reasons for Polytobacco Use

After reviewing the literature, an expert panel including the authors of the current paper and colleagues in the area of tobacco use, particularly ATP use, developed a list of potential reasons for polytobacco use (Table 2). Participants were asked, “How true for you is each possible reason for using more than one tobacco product in the past 4 months?” with response options of 0=not at all true to 6=extremely true.

#### Sociodemographics

We assessed age, sex, race, and ethnicity.

#### Tobacco Use

Participants were first asked to report the number of days they used each tobacco product – cigarettes; little cigars or cigarillos (such as Swisher Sweets or Black n Milds); smokeless tobacco (such as chew, snus, or dissolvable tobacco); e-cigarettes (such as Blu or Njoy); hookah – in the past 4 months (to cover the duration of time between each wave of assessment). Those that reported any use in the past 4 months were then asked to report the number of days they used the respective tobacco product. Pictures were presented alongside each to aid in participant understanding. Tobacco use was dichotomized as any versus no use in the past 30 days, as preliminary analyses indicated similar results using these as continuous variables.

#### The Hooked on Nicotine Checklist

The Hooked on Nicotine Checklist^45, 46^ is a reliable and valid measure of diminished autonomy over tobacco. It is uniquely suited for use with smokers whose cigarette consumption is low. The verbiage of this scale was adapted in the current study to be applicable to all types of tobacco and nicotine product use (i.e., not only cigarette smoking; e.g., “Have you ever tried to quit using tobacco or nicotine, but couldn't?”). Cronbach's alpha for this scale in this study was .94.

#### The Motives for Smoking Scale

The Motives for Smoking Scale^47, 48^ assesses the extent to which each of 15 smoking-related motives is true for a participant (1=not at all true to 5=very true). The measure contains questions about four common motives: social (4 items, e.g., “Smoking helps you fit in with other people”), self-confidence (4 items, e.g., “Smoking makes you feel more self-confident”), boredom relief (2 items, e.g., “Smoking is something to do when you're bored”), and affect regulation (5 items, e.g., “Smoking helps you calm down when you're feeling tense or nervous,” “Smoking cheers you up when you're in a bad mood”). Higher scores indicate that the motive is more relevant. The verbiage of this scale was adapted in the current study to be applicable to all types of tobacco and nicotine product use (e.g., “Using tobacco or nicotine helps you fit in with other people”). In the current study, alphas for the social subscale, the self-confidence subscale, the boredom relief subscale, and the affect regulation subscale were .89, .86, .94, and .92, respectively.

#### Perceptions of Tobacco Products

We also asked about perceptions of tobacco products (cigarettes, LCCs, SLT, e-cigarettes, hookah) on a Likert scale of 1=not at all to 7=extremely. This included perceptions about addictiveness of each tobacco product, harmfulness of product use, and social acceptability of each product^32^.

#### Readiness to Quit

Among past 4-month users of any tobacco product, participants were asked, “What best describes your intentions regarding quitting the use of all nicotine and tobacco: never expect to quit; may quit in the future, but not in the next 6 months; will quit in the next 6 months; and will quit in the next month”^49^. For the present study, this variable was dichotomized as intending to quit all tobacco products in the next 30 days versus all other responses.

#### Social Factors

We asked if a parent currently used each tobacco product^32^ and the number of five closest friends using each tobacco product^32^. These items were operationalized as dichotomous variables (e.g., at least one friend used versus none).

#### Other Substance Use

Participants were first asked to report the number of days they used alcohol and marijuana, respectively. Those that reported any use in the past 4 months were then asked to report the number of days they used the respective product. Given the distributions of use, alcohol use was used as a continuous variable, while marijuana use was used as a dichotomous variable.

#### Depressive Symptoms

We assessed depressive symptoms using the Patient Health Questionnaire – 9 item (PHQ-9)^50^. We developed a continuous variable resulting from a sum of the items. Cronbach's alpha in the current study was .86.

### Data Analysis

We conducted a factor analysis of the Reasons for Polytobacco Use items using Promax rotation. We used eigenvalues of greater than 1 as the criteria for number of factors. Then, we examined the content and internal consistency of the factors. Descriptive statistics regarding sociodemographics, tobacco use characteristics, and factors aimed at examining convergent and discriminant validity were calculated. We then conducted bivariate analyses examining subscale scores in relation to these correlates of interest. Analyses were conducted in SPSS 23.0, and alpha was set at .05.

## RESULTS

### Participant Characteristics

Our sample was an average age of 20.40 (SD=1.84), 48.0% male, 64.8% White, 21.9% Black, and 8.1% Hispanic (Table 1). Our sample included 40.9% cigarette users, 30.4% LCC users, 13.5% SLT users, 31.3% e-cigarette users, and 30.7% hookah users. On average, participants used 2.47 (SD=1.24) types of tobacco products in the past 30 days. Average number of days of alcohol use was 6.53 (SD=6.82), and 33.0% of our sample reported using marijuana in the past 30 days.

### Factor Analysis

Factor analysis identified four factors (see Table 2): 1) Instrumentality, which indicated a range of functions polytobacco use served related to physical sensations (e.g., buzz, enhancing effects of other tobacco products or other substances), appetite reduction, and other substance use reduction; 2) Social Context, indicating use of different products in different social contexts or in relation to self-presentation; 3) Displacement, indicating use of one tobacco product to reduce or quit the use of another or using one tobacco product to circumvent smoke-free policies; and 4) Experimentation, indicating experimental use of tobacco products but no specific link between the use of the distinct products. Two items were deleted (My friends or family introduced me to a new tobacco product; I use different tobacco products for different reasons, e.g., to concentrate, to relax), as these items were deemed vague and did not assess specific reasons for use. These four factors accounted for 62.5% of the variance. Cronbach's alphas for each subscale were: .91, .78, .82, and .56, respectively. The correlations ranged from .14 between Displacement and Experimentation to .58 between Instrumentality and Displacement (Table 3).

### Convergent and Discriminant Validity

Table 3 provides data regarding convergent and discriminant validity. Instrumentality was associated with current use of each tobacco product; greater number of tobacco products used; higher nicotine dependence; higher motivation subscale scores across motivations (e.g., social, self-enhancement, boredom, affect regulation); friends’ tobacco use; current marijuana use (but not alcohol use); greater perceived addictiveness of cigarettes (r=−.09, p=.033, not in tables); and greater perceived harm to health of cigarette use (r=−.09, p=.029). Social Context was associated with current use of cigarettes, SLT, e-cigarettes, and hookah (but not LCCs); greater number of tobacco products used; higher nicotine dependence; higher motivation subscale scores across motivations; friends’ tobacco use; number of days of alcohol use in the past month (but not marijuana); and greater perceived social acceptability of hookah use (r=.11, p=.008). Displacement was associated with current use of cigarettes, SLT, e-cigarettes, and hookah (but not LCCs); greater number of tobacco products used; higher nicotine dependence; higher motivation subscale scores across motivations; alcohol use (but not marijuana); greater perceived harm to health of e-cigarette use (r=−.09, p=.039); and greater perceived social acceptability of cigarette and e-cigarette use (r=.10, p=.025 and r=.13, p=.002, respectively). Experimentation was associated with current use of e-cigarettes (but no other tobacco product); higher motivation subscale scores across motivations; friends’ tobacco use; alcohol use (but not marijuana); greater perceived addictiveness of cigarettes, LCCs, SLT, and e-cigarettes (r's ranging from .10 for e-cigarettes to .19 for cigarettes, p's<.05); greater perceived harm to health of cigarettes, LCCs, and SLT (r's ranging from .13 for e-cigarettes to .22 for cigarettes, p's<.01); and greater perceived social acceptability of use of each product (r's ranging from .10 for SLT to .21 for hookah, p's<.05).

Most noteworthy are the associations or lack of associations that discriminate the subscales. Experimentation was not associated with current use of any tobacco product with the exception of e-cigarettes, nor was it associated with nicotine dependence; however, Experimentation was associated with greater perceived addictiveness and harm of using tobacco products as well as greater perceived social acceptability of tobacco use. Displacement and Social Context showed similar associations with the exception that Social Context was associated with having friends who used tobacco while Displacement was not. Finally, Instrumentality and Social Context were quite similar with the exception of Instrumentality not being associated with alcohol use. Interestingly, Instrumentality was the only factor associated with current marijuana use. It is also noteworthy that sociodemographics, readiness to quit using tobacco and nicotine, parental use of tobacco, and depressive symptoms were not significantly associated with scores on any of the Reasons for Polytobacco Use Scale subscales.

## DISCUSSION

There is a dearth of research, particularly quantitative research, on reasons for polytobacco use patterns among young adults. This is critical, as using tobacco during this developmental period may lead to establishment of chronic tobacco use and addiction, ultimately increasing the risk of various diseases and illnesses. Interventions aimed at addressing tobacco use among young adults must be informed by a solid understanding of different use profiles and the reasons young adults use tobacco and engage in polytobacco use. This is particularly important in this population given the significant proportion of young adult tobacco users who are polytobacco users^16, 17^. At Wave 3, nearly half of all tobacco users used more than one tobacco product in the past 4 months, with over half of polytobacco users using 3 or more different tobacco products. Thus, polytobacco use is a significant issue to address within this population. This study provides a preliminary scale that could be used to assess reasons for polytobacco use.

Aligned with the Theory of Planned Behavior^35, 36^ and Social Cognitive Theory^37^, this study indicated that four categories of reasons for polytobacco use were identified: Instrumentality, Social Context, Displacement, and Experimentation. Instrumentality and Displacement largely reflected outcome expectancies and attitudes towards tobacco use, Social Context reflected the importance of social influences and image maintenance, and Experimentation seemed to reflect both attitudes toward tobacco products as well as social influences. Each of the subscales was correlated with one another, with the weakest correlations being found between Experimentation and each of the others, particularly Displacement. This indicates, as we might expect, that Experimentation as a reason for polytobacco use is highly distinct from the other reasons. This is further indicated by the fact that Experimentation demonstrated the most unique profile in terms of convergent and discriminant validity. For example, Experimentation was only associated with the use of e-cigarettes but not the other products, nor was it associated with symptoms of nicotine dependence. While Experimentation was associated with each of the motivations indicated by the Motives for Smoking Scale (e.g., social, self-enhancement, boredom, affect regulation), it had the lowest correlations with each among all of the subscales of the Reasons for Polytobacco Use Scale. In addition, higher Experimentation scores was associated with perceiving the various tobacco products to be more addictive and harmful to one's health, with only a couple of correlations between these measures and other subscales. Collectively, these findings might suggest that those polytobacco users who endorse using for experimentation are less motivated by social, self-enhancement, boredom, or affect regulation and are aware of the risks of tobacco use. Interestingly, Experimentation was also associated with greater perceived social acceptability, as well as having friends who use tobacco. Thus, their social context might be playing a role in their perceptions about tobacco use as well as be a source of tobacco products with which to experiment^32, 38^.

Social Context and Displacement were correlated with one another. Accordingly, their associations with many factors were quite similar. Displacement had the highest correlations with use for boredom and affect regulation per the Motives for Smoking Scale, while Social Context had the highest correlation with using for social reasons. Moreover, Social Context was associated with having friends who use tobacco and greater perceived social acceptability of hookah use (which aligns with prior research^32, 38^), while Displacement was associated with greater perceived social acceptability of cigarette and e-cigarette use. This is intuitive, particularly given that the correlation between Displacement and nicotine dependence was much higher than the correlation between Social Context and nicotine dependence scores. This might suggest that those endorsing use dictated by Social Context might have a more malleable use pattern that does not require them to substitute products when they cannot use tobacco or in efforts to quit or reduce the use of one tobacco product. On the other hand, those endorsing Displacement-related reasons may be dealing with addiction through substituting one product for another or by using one product to quit or reduce use. These are commonly endorsed reasons for switching products or using products simultaneously^33, 34^. This aligns with the finding that perceived harm of e-cigarette use was associated with higher Displacement scores. Also note that cigarette use prevalence – as well as prevalence of SLT and e-cigarette use (the ATPs most likely used to displace cigarette use) – was most highly correlated with Displacement.

Finally, Instrumentality was correlated highly with both Social Context and Displacement, indicating that those endorsing these other two reason categories also used various tobacco products to achieve certain outcomes. Thus, Instrumentality demonstrated similar associations with other measures as Social Context and Displacement. However, Instrumentality was the only factor associated with current marijuana use and with current use of LCCs. This might suggest a specific profile of young adults who are likely concurrently using tobacco products and marijuana, as LCC and marijuana co-use is a use profile frequently identified among young adults^16, 51, 52^. This use profile seems to indicate using tobacco products to achieve certain outcomes – achieving a buzz, increasing the effects of one another and other drugs (particularly marijuana), or substituting tobacco for alcohol and other drugs. Relatedly, Instrumentality had the highest correlation with using for self-enhancement and also was the only factor associated with lower perceived addictiveness and harm of cigarettes. Thus, those endorsing Instrumentality may be at particularly high risk for continued tobacco use and may develop greater nicotine addiction.

It is noteworthy that parental use of tobacco, depressive symptoms, and readiness to quit using tobacco and nicotine were not associated with scores on any of the Reasons for Polytobacco Use Scale subscales. Parental use may influence use patterns less during this phase of life, particularly among college students who are immersed in college life and more influenced by peers^32^. Parental use may also be mediated by another more proximal variable. While depressive symptoms per say were not associated with the distinct subscale scores, using for affect regulation motives was, particularly with Displacement reasons for polytobacco use, which was also highly associated with nicotine dependence. This might potentially indicate that those using tobacco products during this transitory part of life to regulate negative emotions may be at risk for increased depressive symptoms later in life. Finally, the null findings for readiness to quit using tobacco or nicotine may reflect the phenomenon that many young adult users, particularly nondaily tobacco users, may not perceive themselves as “smokers” and thus not perceive a need to “quit” using^53^.

The current study has implications for research and practice. Future research might leverage these findings to develop cessation or prevention interventions that address the range of reasons for tobacco and polytobacco use in young adults. Additionally, examination of reasons for concurrent use of tobacco and marijuana might be important, given the high concurrent use rates of these products^54, 55^. In practice, health educators and clinicians must be aware of the high polytobacco use rates among young adults and the different user profiles in order to effectively identify young adult polytobacco users and intervene to address these reasons for use.

### Limitations

This study has some limitations. First, the study sample was drawn from colleges/universities in Georgia, is subject to selection bias, and may not generalize to all young adults. However, our sample is diverse in terms of race/ethnicity, geographic location (urban vs. rural), and socioeconomic backgrounds. Second, our scope of items may not be inclusive of all potentially important reasons for polytobacco use; however, the items developed and included here were drawn from the literature related to tobacco or polytobacco use in this population. Third, the cross-sectional design does not allow us to draw causal attributions or determine intra-individual trajectories of substance use over time. These analyses are also limited by the self-report nature of the assessments. Additionally, whether or not the participant was aware of the substance use of others (e.g., parents or friends) is a limitation of the data.

## CONCLUSIONS

This study highlighted the prevalence of polytobacco use in young adults, as nearly half of all tobacco users used more than one tobacco product in the past 4 months, with nearly half of the polytobacco users using 4 or more different tobacco products. The current study addressed a gap in the literature, specifically regarding how to quantitatively assess and characterize reasons for polytobacco use. Four factors indicated distinct reasons for use: Instrumentality, Social Context, Displacement, and Experimentation. Each of these factors demonstrated unique convergent and discriminant validity. The use of this scale to characterize polytobacco using young adults may help inform and target interventions aimed at addressing tobacco and polytobacco use among young adults. 

## Figures and Tables

**Table 1:** Participant sociodemographic and tobacco use characteristics, n=540

Variable	M (SD) or N (%)
**Sociodemographics**	
**Age (SD)**	20.40 (1.84)
**Sex (%)**	
**Female**	281 (52.0)
**Male**	259 (48.0)
**Race/Ethnicity (%)**	
**White**	350 (64.8)
**Black**	118 (21.9)
**Asian**	31 (5.7)
**Other**	41 (7.6)
**Hispanic (%)**	44 (8.1)
**School Type (%)**	
**Public university**	174 (32.2)
**Private college/university**	190 (35.2)
**HBCU**	69 (12.8)
**Technical college**	107 (19.8)
**Tobacco-Related Measures**	
**Current tobacco use (%)**	
**Cigarettes**	221 (40.9)
**LCCs**	164 (30.4)
**SLT**	73 (13.5)
**E-cigarettes**	169 (31.3)
**Hookah**	166 (30.7)
**Number of products used (SD)**	2.47 (1.24)
**Nicotine dependence a (SD)**	2.06 (3.21)
**Social motives b (SD)**	7.15 (3.56)
**Self-enhancement motives b (SD)**	6.23 (3.33)
**Boredom motives b (SD)**	3.84 (2.40)
**Affect regulation motives b (SD)**	10.25 (5.69)
**Social Factors**	
**Parental use of tobacco (%)**	209 (38.7)
**Friend use of tobacco (%)**	491 (90.9)
**Substance Use & Mental Health**	
**Number of days alcohol use, past 30 days (SD)**	6.53 (6.82)
**Any marijuana use, past 30 days (%)**	178 (33.0)
**Depressive symptoms c (SD)**	16.91 (90.02)

a **Per the Hooked on Nicotine Checklist**

b **Per the Motives for Smoking Scale**

c **Per the PHQ-9**

**Note: Perceived addictiveness, harm to health, and social acceptability not reported in tables, as this includes 15 variables (3 dimensions × 5 tobacco products).**

**Abbreviations: HBCU = Historically Black College or University; LCC = little cigars or cigarillos; SLT = smokeless tobacco.**

**Table 2:** Reasons for Polytobacco Use Scale items and factor loadings

	Component			
	1	2	3	4
**Instrumentality**				
**Using different products together provides a great buzz.**	.844	.111	−.136	.047
**I get a different kind of buzz from different tobacco products.**	.478	.302	−.016	.095
**I get sick of the taste of some tobacco products but enjoy the buzz, so I use other tobacco products sometimes.**	.626	.162	.027	−.007
**I use some tobacco products to increase the effects of other tobacco products.**	.881	−.021	−.009	−.059
**I use some tobacco products to increase the effects of alcohol.**	.524	.412	−.239	−.034
**I use some tobacco products to increase the effects of illicit drugs.**	.712	.233	−.201	−.060
**I use some tobacco products to increase the effects of prescription drugs.**	.794	−.083	.117	−.033
**I was using more than one tobacco product to help me reduce my appetite.**	.677	−.128	.278	.007
**I was using more than one tobacco product to help me reduce my use of alcohol.**	.786	−.156	.223	−.026
**I was using more than one tobacco product to help me reduce my use of other drugs.**	.827	−.149	.158	.039
**Social Context**				
**I use different tobacco products around different people (friends, peers at school, family).**	−.125	.891	.081	−.115
**I use different tobacco products in different places (home, school, work, bars, parties).**	−.030	.869	.103	−.133
**I use tobacco product others are using to be sociable.**	.126	.593	−.038	.122
**I'm ok with the image of me that using some tobacco products reflects, but not others.**	.178	.403	.049	.159
**Displacement**				
**I regularly use one tobacco product but only experimented with the other tobacco products.**	−.147	.335	.400	.281
**I was using one tobacco product to try to quit using another tobacco product.**	.044	.023	.874	−.058
**I was using one tobacco product to try to reduce my use of other tobacco products.**	.010	.026	.917	−.066
**I was using more than one tobacco product because I couldn't smoke (cigarettes, cigars, etc.) in some places or at some times.**	.224	.134	.583	−.017
**Experimentation**				
**The use of one tobacco product had nothing to do with the use of the other.**	−.156	.076	−.053	.772
**I just like to experiment with different tobacco products.**	.128	.045	.166	.589
**I only experimented with these products but did not use any of them regularly.**	.044	−.189	−.148	.811

**Note: Extraction Method: Principal Component Analysis. Rotation Method: Promax with Kaiser Normalization. Rotation converged in 6 iterations.**

**Note: Cronbach's alpha for Instrumentality, Social Context, Displacement, and Experimentation were: 90, .91, .78, .77, and .56, respectively. Average subscale scores were: Instrumentality 1.54 (SD=0.87); Social Context 2.00 (SD=1.15); Displacement 1.69 (SD=1.04); and Experimentation 2.87 (SD=1.41).**

**Table 3:** Correlations among Reasons for Polytobacco Use subscales and related factors

Variable	Instrumentality	Social Context	Displacement
**Reasons for Polytobacco Use**			
**Instrumentality**	--	.53^***^	.58^***^
**Social Context**		--	.38^***^
**Displacement**			--
**Experimentation**			
**Tobacco-Related Measures**			
**Current Tobacco Use**			
**Cigarettes^ǂ^**	.10^*^	.10^*^	.30^***^
**LCCs^ǂ^**	.12^**^	.03	.05
**SLT^ǂ^**	.10^*^	.13^**^	.15^**^
**E-cigarettes^ǂ^**	.10^*^	.13^**^	.19^***^
**Hookah^ǂ^**	.18^***^	.11^*^	.10^*^
**Number of products used**	.22^***^	.18^***^	.29^***^
**Nicotine dependence^a^**	.18^**^	.09^*^	.46^***^
**Social motives^b^**	.42^***^	.44^***^	.38^***^
**Self-enhancement motives^b^**	.51^***^	.39^***^	.48^***^
**Boredom motives^b^**	.39^***^	.32^***^	.48^***^
**Affect regulation motives^b^**	.40^***^	.28^**^	.49^***^
**Readiness to quit all tobacco^ǂ^**	.04	-.02	-.01
**Readiness to quit all tobacco^ǂ^**			
**Social Factors**			
**Parental use of tobacco^ǂ^**	-.04	-.03	.01
**Friend use of tobacco^ǂ^**	.11^*^	.09^**^	.06
**Substance Use & Mental Health**			
**Number of days of alcohol use, past 30 days**	.05	.13^**^	.10^*^
**Any marijuana use, past 30 days^ǂ^**	.14^**^	.08	.06
**Depressive symptoms^c^**	.03	-.03	.02

ǂ **Point biserial correlations; all others are Pearson correlations.**

**Note:**

* **< .05**

** **< .01**

*** **< .001**

a **Per the Hooked on Nicotine Checklist**

b **Per the Motives for Smoking Scale**

c **Per the PHQ-9**

**Abbreviations: HBCU = Historically Black College or University; LCC = little cigars or cigarillos; SLT = smokeless tobacco.**

**Note: Perceived addictiveness, harm to health, and social acceptability not reported in tables, as this includes 15 variables.**
